# Integrated Resonant Micro/Nano Gravimetric Sensors for Bio/Chemical Detection in Air and Liquid

**DOI:** 10.3390/mi12060645

**Published:** 2021-05-31

**Authors:** Hao Jia, Pengcheng Xu, Xinxin Li

**Affiliations:** State Key Lab of Transducer Technology, Shanghai Institute of Microsystem & Information Technology, Chinese Academy of Sciences, 865 Changning Road, Shanghai 200050, China; hao.jia@mail.sim.ac.cn (H.J.); xpc@mail.sim.ac.cn (P.X.)

**Keywords:** integrated resonators, gravimetric sensors, bio/chemical sensing, quality factor, resonant modes

## Abstract

Resonant micro/nanoelectromechanical systems (MEMS/NEMS) with on-chip integrated excitation and readout components, exhibit exquisite gravimetric sensitivities which have greatly advanced the bio/chemical sensor technologies in the past two decades. This paper reviews the development of integrated MEMS/NEMS resonators for bio/chemical sensing applications mainly in air and liquid. Different vibrational modes (bending, torsional, in-plane, and extensional modes) have been exploited to enhance the quality (*Q*) factors and mass sensing performance in viscous media. Such resonant mass sensors have shown great potential in detecting many kinds of trace analytes in gas and liquid phases, such as chemical vapors, volatile organic compounds, pollutant gases, bacteria, biomarkers, and DNA. The integrated MEMS/NEMS mass sensors will continuously push the detection limit of trace bio/chemical molecules and bring a better understanding of gas/nanomaterial interaction and molecular binding mechanisms.

## 1. Introduction

The past two decades have witnessed significant development in sensor technologies for the recognition and detection of chemical (e.g., volatile organic compounds, VOCs) and biological species (e.g., cells, proteins) in ambient and liquid environments. In contrast to the optical methods, micro-/nanoelectromechanical systems (e.g., microcantilevers), featured by miniature device size, exquisite detection limit (from part per million to part per trillion, or micromolar to femtomolar), and easy to be on-chip integrated, have offered better opportunities to detect the trace analytes and capture the molecular interaction processes in gas and liquid phases [1,2,3].

Taking the prevailing microcantilever sensors as an example, two different operational modes (i.e., static and dynamic modes) have been engineered for bio/chemical sensing. In static mode, the cantilever surface is functionalized to have a good affinity to the target molecules. The stress change at the cantilever surface (owing to electrostatic repulsion or attraction, steric interactions, hydration, and entropic effects) causes the cantilever bending, which is usually measured by an optical lever system [4]. Since the responses of surface-stress sensors are difficult to interpret, a dynamic scheme has been proposed by operating a cantilever at resonance. A tiny mass loading (Δ*m*) at a cantilever free end can lead to a resonant frequency downshift (Δ*f*), which defines the mass sensitivity: ℜ=Δf/Δm=−f/2M, where $f=1/2\pi \sqrt{k/M}$ is the resonant frequency, *k* and *M* are the effective spring constant and effective mass of the resonant device [5]. Therefore, one can perceive that micro-and nanoelectromechanical (MEMS/NEMS) resonators promise gravimetric detection of trace analytes down to a fraction of the sensor mass. In comparison with the conventional quartz crystal microbalances (QCM), the silicon micro-machined resonant cantilevers possess finer mass resolution, smaller size, low-cost batch fabrication, and easy IC-compatible integration [6]. Although pioneering efforts have been made towards attogram and atomic-level resonant mass sensing, with outstanding device performance often achieved by off-sensor optical detection methods in high vacuum or at low temperature [7,8,9,10,11,12,13,14,15,16], there is an increasing need for potable micro- and nano-gravimetric sensors with integrated resonance excitation and readout elements on-chip, toward real-world bio/chemical sensing applications.

To date, several on-chip resonance excitation and readout mechanisms have been developed, considering their compatibility with ambient and liquid conditions (especially conductive biosolutions). As to resonance readout, most microcantilevers use piezoresistive effect, as the doped semiconducting materials (mostly silicon) provide very large gauge factors, and the fabrication process has been well-developed and optimized [17,18]. The piezoresistors are often patterned at the device’s clamping points (e.g., fixed end of the cantilever), where the largest stress is expected at resonance. Wheatstone bridge with 4 terminal piezoresistors is also desirable to improve the displacement sensitivity to nanometer scale in air and liquid.

As to resonance excitation, electrothermal excitation has been widely adopted on many resonant MEMS/NEMS prototypes, such as microcantilevers [19], ‘dog-bone’ resonators [20], and disk resonators [21]. A DC + AC voltage signal is typically applied to a heating resistor patterned at the device’s clamping points, and the time-varying heating power whose cycle matches the device’s resonant frequency induces the mechanical vibration. On the other hand, electromagnetic excitation has been achieved with metal loops patterned around the cantilevers. With the presence of an external magnetic field (tens of mT) and an AC electrical current flowing through the metal loops, Lorentzian force drives the bending or torsional vibration, depending on the positions of the metal loops on the resonators [17,22].

Alternatively, the piezoelectric approach emerges with the successful synthesis of ceramics with high piezoelectric constants (such as aluminum nitride, AlN, Pb-based lanthanum-doped zirconate titanates, PZT). The piezoelectric effect enables simultaneous self-excitation/readout. A pure AC input signal creates time-varying stress at the clamping points, while another AC piezoelectric current signal is generated to read out the resonant frequency [23,24,25].

**Figure 1:**
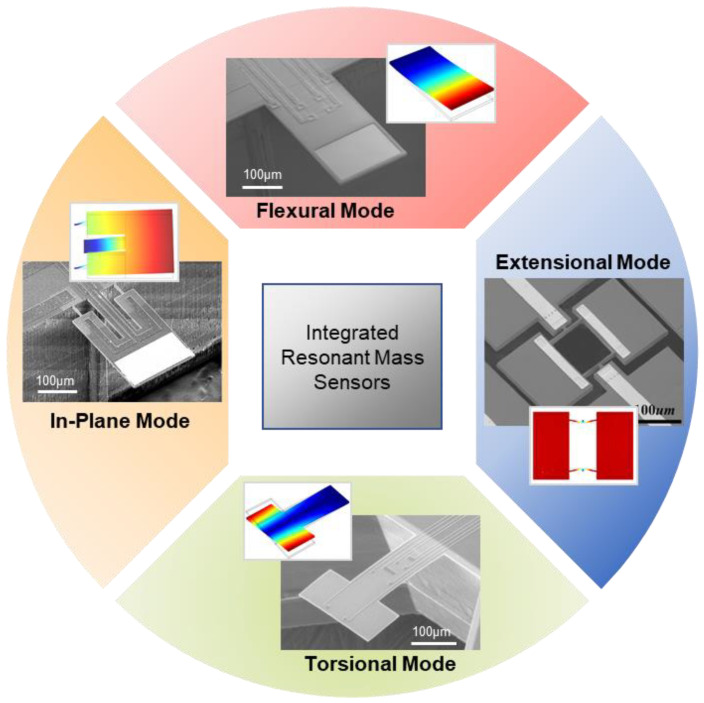
Integrated resonant gravimetric sensors using different vibrational modes, such as bending, torsional, in-plane, and extensional modes. Reprinted with permission from [17,19,20,26]. Copyright 2007 American Institute of Physics, 2019 Royal Society of Chemistry, 2016 IEEE, 2011 Elsevier B.V.

Although on-chip, all-electrical integration brings immense simplification to the bulky, expensive measurement system that is inevitable for optical readout, researchers are still confronted with technical challenges of operating resonant sensors in viscous media that dissipates resonating energy, and great endeavors have been made to achieve better device performance (e.g., higher *Q*, better mass sensitivity and mass resolution) in air and liquid by studying different vibrational modes, such as bending, torsional, in-plane, and extensional modes (as shown in Figure 1 and Table 1).

## 2. Integrated Resonant Gravimetric Sensors Using Different Vibrational Modes

### 2.1. Integrated Resonant Gravimetric Sensors Using Fundamental Bending Modes

**Figure 2:**
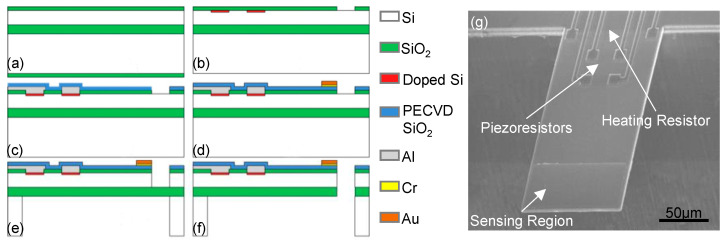
Bending-mode cantilever mass sensors. (**a**–**f**) Fabrication process of microcantilevers showing on-chip integration of electrothermal excitation and piezoresistive readout. (**g**) Typical resonant sensor with sensing region at the free end for nanomaterial loading. Reprinted with permission from [18]. Copyright 2009 IOP Publishing Ltd.

Microcantilever vibrating at its fundamental bending mode is the most common type of resonant mass sensor. Figure 2b shows a typical cantilever mass sensor with integrated thermoelectric excitation and Wheatstone bridge readout components [18]. As illustrated in Figure 2a, 4 piezoresistors and 1 heating resistor near the fixed end of the cantilever are created by boron-doping through thermally grown silicon oxide (SiO_2_) windows. With patterned aluminum interconnects covered by PECVD SiO_2_ passivation layer and Au/Cr sensing pad at the free end, the cantilever is finally released by the backside deep reactive ion etching (DRIE), followed by the removal of SiO_2_ by hydrofluoric acid (HF). The heating power with a DC + AC voltage is given by $P={\left(V_{DC}+V_{AC}\cos\omega t\right)}^{2}/R$, where *R* is the resistance of the piezoresistive heater, ω=2πf, where *f* is the driving frequency. One can perceive that the mechanical resonance is excited by the $V_{DC}\cdot V_{AC}\cos\omega t/R$ component (given that $V_{DC}>>V_{AC}$). The sensing region at the cantilever free end is covered by an Au film for subsequent chemical functionalization. Such resonant cantilevers with dimensions of 200 × 100 × 3 μm have exhibited fundamental-mode resonant frequencies *f* ~100 kHz, and quality factors *Q* > 100. With a mass sensitivity ~1.5 Hz/pg and mass resolution ~0.1 pg, various hazardous chemical vapors, e.g., Trinitrotoluene (TNT), dimethyl methyl phosphonate (DMMP), VOCs and biological species (e.g., bacteria, proteins, DNA) with very low concentrations have been successfully detected.

It is worth noting that with all-electrical integration, a resonator is readily connected to a phase-lock loop (PLL) for real-time frequency tracking. Hence, the minimum detectable mass is defined by the minimum detectable phase change of the system, given by δm=(δθ/Q)M [30]. Therefore, the mass resolution is significantly dependent on the *Q* factor, which is inevitably deteriorated by the strong viscous damping in air and liquid. Therefore, researchers have been exploring other high-order resonance modes to improve the *Q* factor and mass sensing performance.

### 2.2. Integrated Resonant Gravimetric Sensors Using Higher-Order Bending Modes

The enhancement in *Q* factor and mass sensing performance when using high-order flexural modes has been demonstrated in many cases [9,27,31,32,33,34,35]. Taking cantilever resonators in air as an example, a ~5-fold increase in *Q* factors, ~6-fold increase in mass sensitivity, and >8-folder improvement in mass resolution have been demonstrated using the 2nd-order bending mode than using fundamental mode (as shown in Table 1). The increase in mass sensitivity (from 0.43 to 2.7 Hz/pg) can be attributed to the higher resonant frequency of 2nd-order bending mode (~298 kHz), which is ~6-folder higher than that of the fundamental mode (~47.8 kHz). The improvement in *Q* factor (from 867 to 168) has been analyzed from the perspective of flow pattern over the width of the cantilever. Using finite element simulation, less energy dissipations into the viscous media have been visualized for high order bending modes, leading to higher *Q*s [28,36,37].

### 2.3. Integrated Resonant Gravimetric Sensors Using Torsional Modes

**Figure 3:**
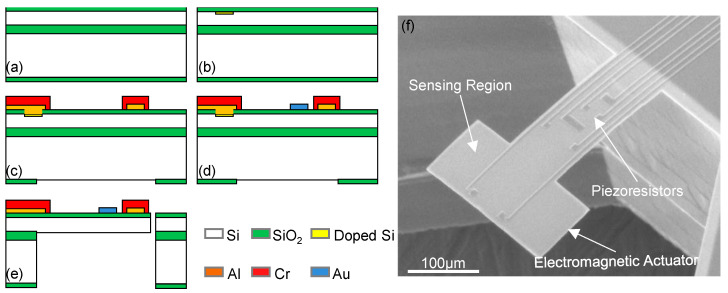
Torsional-mode cantilever mass sensors. (**a**–**e**) Fabrication process of the microcantilevers showing on-chip integration of electromagnetic excitation and piezoresistive readout. (**f**) Typical resonant sensor with 2 sensing regions for nanomaterial loading. Reprinted with permission from [17]. Copyright 2007 American Institute of Physics.

Torsional modes have also been exploited to improve the sensing performance of resonant mass sensors [17,22,28,38]. Figure 3b shows a typical T-shaped torsional-mode cantilever with on-chip integrated electromagnetic excitation and piezoresistive readout components [17]. The fabrication process of torsional-mode resonators is similar to that of bending-mode resonators. 4 piezoresistors are formed by boron-doping using SiO_2_ mask. Aluminum is patterned not only as the interconnects but also as the metal loop for electromagnetic excitation. After the Cr/Au sensing pads are defined by liftoff, the device is released by both frontside RIE, backside DRIE, and SiO_2_ removal by HF. Together with a decent increase in *Q* factor, Xia et al. have reported a significant enhancement in mass sensing performance using torsional modes. The >10-fold increase in mass sensitivity is attributed to the much higher resonant frequencies of the torsional modes than that of the fundamental mode. Benefited from high-order torsional vibrations, the mass detection limit has been improved by almost 30 times, down to 9 fg in air [22].

### 2.4. Integrated Resonant Gravimetric Sensors Using In-Plane Modes

**Figure 4:**
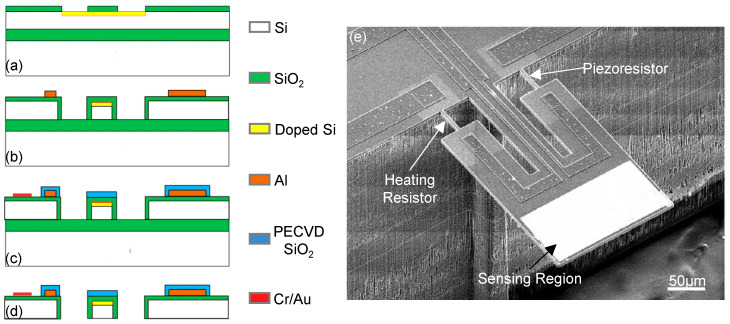
In-plane-mode resonant microcantilever mass sensors. (**a**–**d**) Fabrication process of microcantilevers showing on-chip integration of electrothermal excitation and piezoresistive readout. (**e**) Typical resonant sensor with sensing region for loading functional nanomaterials. Reprinted with permission from [26]. Copyright 2011 Elsevier B.V.

Resonant gravimetric sensors using in-plane modes have been studied [26,39,40], as shown in Figure 4b. To achieve effective excitation and detection of in-plane vibration, thin beam legs are doped to form resistors for electrothermal driving and piezoresistive readout. The fabrication process is similar to that for bending-mode cantilevers, as shown in Figure 4a. An additional oxygen annealing step is often performed to protect the vertical sidewalls of the cantilever and tiny beams against electric leakage in conductive solutions since these devices are often designed for bio/chemical sensing in liquid. An important merit of using in-plane mode is the high *Q* factors >2000 in air, which is >10-fold better than that of the fundamental mode (as shown in Table 1). As this type of resonator is often used for biosensing applications in liquid, the sensing performance will be detailed in Section 3.3.

### 2.5. Integrated Resonant Gravimetric Sensors Using Extensional Modes

**Figure 5:**
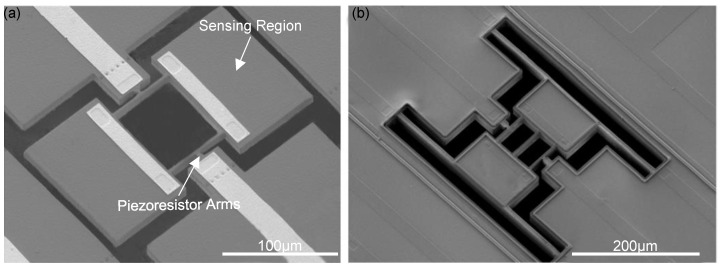
Extensional-mode ‘dog-bone’ mass sensors. (**a**,**b**) Two types of devices with sensing pads connected by dual- and tri-beams. Reprinted with permission from [20]. Copyright 2016 IEEE.

Extensional-mode resonators typically have ‘dog-bone’ structure [20,29,41], which is quite different from a cantilever, as shown in Figure 5. Two large sensing pads are connected by 2 or 3 thin beams and vibrate oppositely along the device length. The piezoresistive arms are designed for electrothermal excitation and piezoresistive readout of the extensional mode. The fabrication process of the ‘dog-bone’ resonator is quite similar to that of in-plane mode cantilevers. Such mass sensors using bulk mode surpass the aforementioned bending, torsional, and in-plane mode resonators in terms of *Q* factor and mass sensitivity. *Q* factors > 11,000 and mass sensitivity up to 10.6 Hz/pg have been reported, which are ~65-time and ~25-time higher than those from fundamental-mode cantilevers (refer to Table 1).

Overall, resonant mass sensors, with integrated excitation and readout schemes and enhanced mass sensing performance by different vibrational modes, have opened up new possibilities for ultrasensitive bio/chemical detection in gas- and liquid phases, which will be detained in the following section.

## 3. Integrated Resonant Gravimetric Sensors for Bio/Chemical Detection

### 3.1. Integrated Resonant Gravimetric Sensors for Gas Detection

The ability to detect trace chemical vapors (e.g., TNT, DMMP), VOCs (e.g., aniline, xylene), and other pollutant gases (e.g., carbon monoxide, CO, sulfur oxide, SO_2_) is highly demanded in environmental protection, industrial pollution control, biomedical systems, and public safety. However, the very low concentration of target gases, interfering gases, and potable sensor design have imposed great challenges in on-the-spot, rapid detection. Therefore, great attention has been made to engineer integrated resonant gravimetric sensors for gas sensing, thanks to their small size, ultra-high sensitivity, and scalability for mass production.

**Figure 6:**
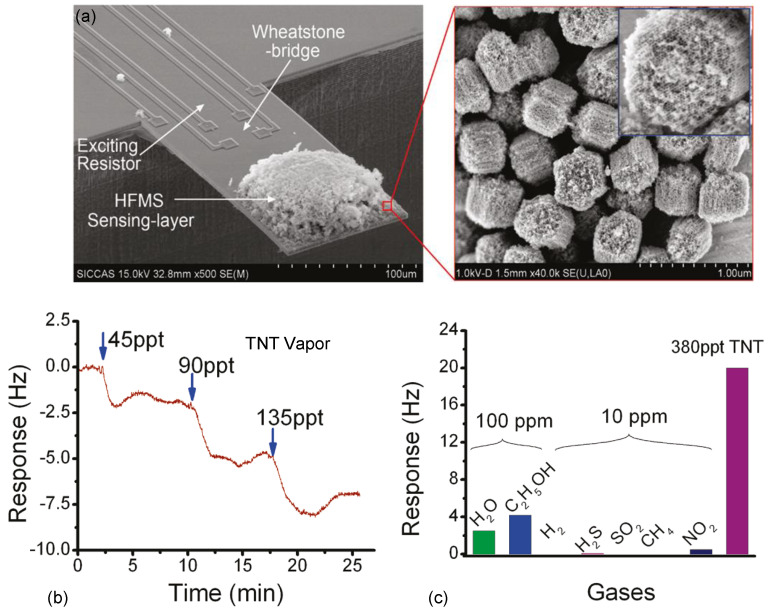
TNT vapor detection using a microcantilever sensor. (**a**) SEM images showing a microcantilever sensor with loaded HEMS nanomaterials. (**b**) Frequency responses of the microcantilever to TNT vapors at different contractions. (**c**) Sensor responses to various kinds of interfering gases compared with 380 ppt TNT vapor. Reprinted with permission from [42]. Copyright 2011 American Chemical Society.

Figure 6a shows an example of a cantilever gas sensor, with nanomaterials (hexafluoro-2-propanol-functionalized mesoporous silica, HFMS, shown in the inset) loaded to the sensing region near the free end [42]. The cantilever is electrothermally excited at its fundamental mode, and the resonant frequency is monitored in real-time using PLL control. When the target gas molecules flow over the cantilever (e.g., 45 ppt, 90 ppt, 135 ppt TNT in Figure 6b), the molecules are adsorbed onto the nanomaterials, hence decrease the resonant frequency. The presence of functionalized nanomaterials can greatly improve the sensor selectivity of the target gas. As shown in Figure 6c, the HFMS-based cantilever resonator is highly responsive to TNT than other interference gases.

So far, resonant cantilever sensors have shown great potential in detecting many kinds of trace analytes, such as chemical vapors, VOCs, and pollutant gases, as summarized in Table 2. In general, the cantilever mass sensors exhibit detection limits from ~100 pb to ~10 ppt level for detecting chemical vapors, such as TNT, DMMA, TMA. The detection limit for VOCs falls in ~1 ppm–100 ppb level, such as aniline, xylene. As to pollutant gases, such as CO, SO_2_, the minimum detectable concentrations as low as ~10 ppb have been demonstrated. The cantilever resonators also promise fast response time from tens of seconds to ~10 min, thanks to the optimized design of resonators and nanomaterials.

### 3.2. Integrated Resonant Gravimetric Sensors for Biosensing in Air

The detection of biological species, such as bacteria, viruses, and proteins is of great importance for disease diagnosis, food safety, and fundamental research. Luckily, some of these species can survive in humid air, hence they can be detected by resonant mass sensors. In these cases, the resonators are operated in ‘dip-and-dry’ mode, without suffering from strong viscous damping. After sufficient time for immobilization of bacteria or antigen-antibody interactions in solution, the resonators are dried, and the resonant frequencies before and after dipping are recorded.

Using such an approach, Xu et al. have reported the detection of Escherichia coli (*E. coli*) O157:H7 down to 10^3^ CFU/mL, and Bacillus Anthracis as low as 10^3^ spores/mL using cantilever mass sensors [57]. With an ultra-high mass resolution down to 9 fg, Xia et al. have demonstrated the detection of 60 ng/mL alpha-fetoprotein (AFP) using torsional-mode resonators [22].

### 3.3. Resonant Gravimetric Sensors for Bio/Chemical Detection in Liquid

Although the ‘dip-and-dry’ method has been proved to be effective for certain cases that avert device immersion in liquid, direct sensing in liquid is highly desirable, especially for biological applications since most biological processes take place in liquid. The much stronger viscous damping in liquid than in air has imposed great challenges in detecting trace bio/chemical analytes in liquid using resonant gravimetric sensors. For example, fundamental-mode cantilever resonators typically exhibit *Q*s ~10–20 when operated in liquid (e.g., water, phosphate buffered saline, PBS) (refer to Table 3). Still, prostate-specific antigen (PSA), C-reactive protein (CRP), DNA with concentrations between 10 μg/mL to 10 ng/mL are detectable using such gravimetric sensors.

**Figure 7:**
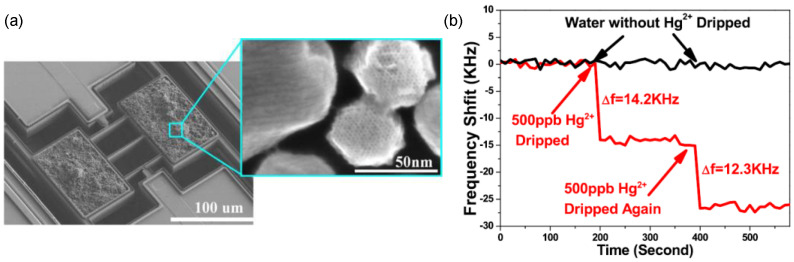
Detection of Hg^2+^ in liquid droplets using a tri-beam extensional-mode resonator. (**a**) SEM images showing a microcantilever sensor with loaded mesoporous silica. (**b**) Frequency responses of the resonator to 500 ppb and 1 ppm Hg^2+^ ion in solution. Reprinted with permission from [20]. Copyright 2016 IEEE.

Several approaches have been taken to improve the sensing performance of resonant mass sensors in liquid. First, as mentioned in Section 2, *Q* factor and mass sensitivity can be improved by exploiting vibrational modes beyond fundamental mode. Figure 7 shows an example of monitoring heavy metal-ion (Hg^2+^) in water (mimicking ion pollution to water resource) using extensional-mode ‘dog-bone’ resonators [20]. *Q* factor ~256 and mass sensitivity ~9.76 Hz/pg have been observed, which are >10-time and ~100-time higher than those of fundamental-mode cantilevers. With –SH modified mesoporous silica loaded to the sensing regions, 500 ppb Hg^2+^ can be easily discerned from frequency response (as shown in Figure 7b). Second, new device structures can be engineered to isolate the resonators from strong liquid damping. For example, suspended microchannel resonators, SMRs [58,59,60,61,62,63,64], have drawn considerable attention because of their unique way of minimizing viscous damping by fabricating fluidic channels inside the cantilevers. Therefore, these devices are operated in vacuum, allowing ultra-high *Q*s on the order of 1000–10,000, and unprecedented mass resolutions < 1 ag. Although this type of device has yet been on-chip fully integrated (resonances are often excited by off-chip piezoshaker, and detected by optical lever), and shown limitations in cases, for example, monitoring adherent mass rather than floating mass, they still hold the records for mass sensing in liquid using cantilevers.

Alternatively, resonant mass sensors can be directly immersed in liquid with decent *Q*s with the help of hydrophobic shells. Such a sensor platform is more suitable for measuring adherent masses, such as adherent cells and molecular binding. Yu et al. have shown the detection of *E. coli.* down to 10^2^ CFU/mL using a parylene-shell encased bending-mode cantilevers [65]. More recently, Wang et al. have extended such a technique to in-plane cantilevers [66]. *Q* factor has been significantly improved to >200, and mass sensitivity has increased to 1.2 Hz/pg. Both are one order of magnitude better than conventional bending-mode cantilevers (refer to Table 3).

## 4. NEMS Resonators for Ultrasensitive Gravimetric Sensing

To continuously pushing the detection limit of the integrated mass sensors, the device sizes have been miniaturized to the nanoscale. For example, nanobeams, nanowires, and nanotubes have shown effective masses reduced by orders of magnitude to attogram. Subsequently, the resonance frequencies have increased by orders of magnitude up to 1 GHz. These devices have been used for atomic and molecular level mass sensing in vacuum, e.g., Au [8], Xe [12,69], Cr [14] atoms, C_10_H_8_ molecule [16], bovine serum albumin (BSA), and β-amylase [15].

However, it worth noting that the limit of detection at single-molecule/atom level has been achieved through operating those NEMS resonators at very-high/ultra-high frequency (VHF/UHF) bands, but in stringent experimental conditions (e.g., high vacuum and low temperature). Meanwhile, the quality (*Q*) factor decreases as the device sizes get smaller, which is due to the increased energy dissipation at the nanoscale than at the microscale.

**Figure 8:**
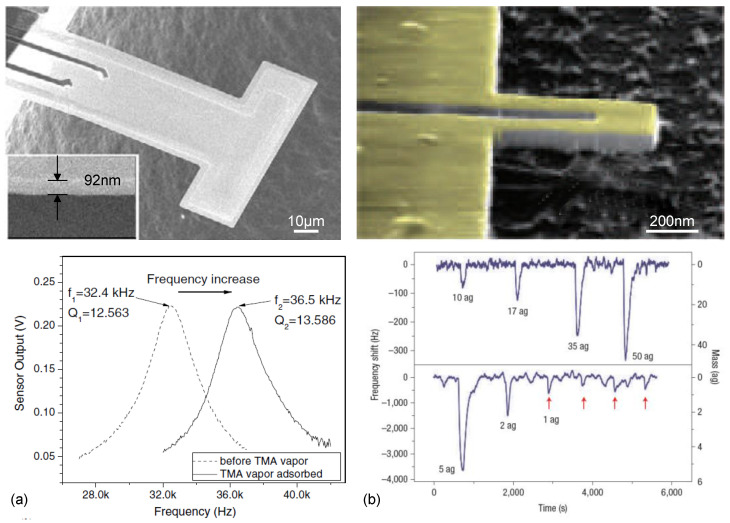
NEMS cantilevers for ultrasensitive gravimetric detection in air. (**a**) A T-shaped nano-thick cantilever with torsional resonant mode ~37.65 kHz exhibits mass sensitivity ~860 Hz/pg, and TMA detection limit <0.1 ppm. (**b**) A SiC nano cantilever with fundamental bending mode in the VHF band (up to 127 MHz), exhibits mass sensitivity ~0.7 Hz/zg, and mass resolution ~0.1 ag. Reprinted with permission from [70,71]. Copyright 2007 Nature Publishing Group, 2010 IOP Publishing Ltd.

Some mass sensing attempts have been made with nanocantilevers in air (as shown in Figure 8), which indeed show exquisite gravimetric sensitivities down to ~0.7 Hz/zg and mass resolution ~0.1 ag [70,71]. More attention still needs to be made to tackle the challenges of detecting VHF/UHF resonances with picometer displacement sensitivity while maintaining sufficient sensing area and decent *Q*s factors in air and liquid, such that the NEMS resonators can be widely used for bio/chemical sensing applications in gas and liquid-phases.

## 5. Conclusions

This paper reviews the development of integrated resonant gravimetric resonators for bio/chemical sensing applications in the past two decades. Bending-, torsional-, in-plane-, and extensional-mode resonators have been studied to enhance the mass sensing performance in viscous media. Thanks to the ultra-high mass sensitivities (typically ~Hz/pg) and mass resolution (pg to fg), trace gas molecules and biological species using integrated resonant mass sensors have been demonstrated. More attention still needs to be made to tackle the challenges of detecting VHF/UHF resonances with picometer displacement sensitivity in air and liquid, such that the nanoresonators (including nanocantilevers and those made of low dimensional materials [14,69,72,73] can be widely used for bio/chemical sensing applications in gas and liquid-phases. In summary, the resonant MEMS/NEMS gravimetric sensors hold promise to continuously push the bio/chemical detection limits and bring a better understanding of gas/nanomaterial interaction and molecular binding mechanisms.

## Figures and Tables

**Table 1:** Performance of typical integrated resonant gravimetric sensors in air using different vibrational modes.

Devices	Resonant Frequency	*Q* Factor	Sensing Performance		Refs
Fundamental Bending-Mode Cantilevers	47.838 kHz	168	0.43 Hz/pg	0.26 pg	[27]
High-Order Bending-Mode Cantilevers	298.132 kHz	867	2.7 Hz/pg	30 fg	[27]
Torsional-Mode Cantilevers	114.805 kHz	252	0.9 Hz/pg	23 fg	[28]
High-Order Torsional-Mode Cantilevers	508.082 kHz	286	5.1 Hz/pg	9 fg	[28]
In-Plane Mode Cantilevers	536 kHz	2096	/	/	[26]
Extensional-Mode Resonators	4.1 MHz	11157	10.617 Hz/pg	0.94 pg	[29]

**Table 2:** Integrated Resonant Gravimetric Sensors for Gas Sensing.

Targets	Sensing Materials	Gas Sensing Performance		Refs
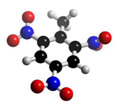 TNT Vapor	HFIP-MWCNTs	~1.2 ppb	~5 min	[43]
	HFIP-Mesoporous Silica	~20.8 ppt	~2 min	[42]
	HFIP-GO/Au- NPs hybrid	~60 ppt	~1 min	[44]
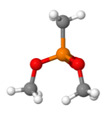 DMMP Vapor	Siloxane-Hyperbranched Polymers	~5 ppb	~3 min	[18]
	Mesoporous Silica Nanoparticles	<1 ppb	<6 min	[45]
	HB-PCSOX-BHPHFB	<300 ppb	5 min	[46]
	BHPF-KIT-5 Mesoporous Silica	~30 ppb	~10 min	[47]
	UiO-66 film	<5 ppb	~10 min	[19]
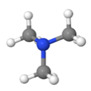 TMA Vapor	−COOH-Mesoporous Silica	~0.8 ppm	~6 min	[48]
	AuNP–rGO	~0.5 ppm	<30 s	[49]
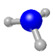 NH_3_ Gas	−COOH-Au-NPs/rGO	<10 ppm	~5 min	[50]
	Carboxyl- Mesoporous Silica Nanoparticles	<5 ppb	~1.8 min	[51]
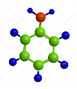 Aniline Vapor	MOF-5	<1.4 ppm	~1.8 min	[52]
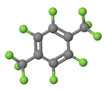 p-xylene	HKUST-1	~120 ppb	~15 min	[53]
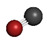 CO	Ni-MOF-74	<10 ppb	~5 min	[54]
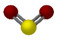 SO_2_	ZnO Nanowires	~70 ppb	~10 min	[55]
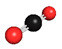 CO_2_ Gas	−NH_2_-MTF	30 ppm	<60 s	[56]

**Table 3:** Integrated Resonant Microgravimetric Sensors for Biological Detection in Liquid.

Device	Dimensions	*f*	*Q*	Sensing Performance		Refs
PZT cantilever	300 × 100 × 0.65 μm	30.95 kHz	-	-	1–100 ng/mL PSA	[23]
Si Cantilever	150 × 140 × 8.2 µm	200 kHz	10	0.02 Hz/pg	10–100 ng/mL PSA	[67]
PZT Cantilever	500 × 500 × 35 μm	36.11 kHz	15–25	0.118 Hz/pg	10 µg/mL CRP	[24]
Si Cantilever	150 × 140 × 8.2 µm	250 kHz	20	0.1 Hz/pg	10–100 ng/mL PSA	[68]
PZT Cantilever	500 × 500 × 32 μm	59 kHz	20	-	100 ng/mL CRP 5 µM ssDNA	[25]
In-Plane Mode Cantilever	190 × 310 × 5 μm	406 kHz	14	8.8 Hz/pg	2 × 10^3^ CFU/mL *E. Coli* EcoRV-enzyme digestion of dsDNA	[26,39]
Rotational Disk	*d* = 500 μm	3.44 MHz	20–80	-	Hybridization between ssDNA (1.0 μM) and HS-ssDNA (2.0 μM)	[21]
Encased Bending-Mode Cantilever	200 × 100 × 3 μm	50.615 kHz	23	-	10^2^ CFU/mL E. Coli	[65]
Encased In-Plane Mode Cantilever	190 × 310 × 3.7 μm	576 kHz	208	1.23 Hz/pg	1–10 μM ATP-Aptamer Interaction	[66]
